# BMP signaling in the development and regeneration of tooth roots: from mechanisms to applications

**DOI:** 10.3389/fcell.2023.1272201

**Published:** 2023-09-15

**Authors:** Cangwei Liu, Hao Guo, Ce Shi, Hongchen Sun

**Affiliations:** ^1^ Department of Oral Pathology, Hospital of Stomatology, Jilin University, Changchun, China; ^2^ Jilin Provincial Key Laboratory of Tooth Development and Bone Remodeling, Changchun, China

**Keywords:** tooth root, BMP signaling, stem cells, bio-root, regeneration

## Abstract

Short root anomaly (SRA), along with caries, periodontitis, and trauma, can cause tooth loss, affecting the physical and mental health of patients. Dental implants have become widely utilized for tooth restoration; however, they exhibit certain limitations compared to natural tooth roots. Tissue engineering-mediated root regeneration offers a strategy to sustain a tooth with a physiologically more natural function by regenerating the bioengineered tooth root (bio-root) based on the bionic principle. While the process of tooth root development has been reported in previous studies, the specific molecular mechanisms remain unclear. The Bone Morphogenetic Proteins (BMPs) family is an essential factor regulating cellular activities and is involved in almost all tissue development. Recent studies have focused on exploring the mechanism of BMP signaling in tooth root development by using transgenic animal models and developing better tissue engineering strategies for bio-root regeneration. This article reviews the unique roles of BMP signaling in tooth root development and regeneration.

## 1 Introduction

A tooth is composed of an enamel-covered crown and a cementum-covered root. The tooth root plays a critical role in transmitting occlusal forces through the periodontal ligaments (PDLs) to the jaw bones. While the mechanisms of tooth crown development have been extensively studied, those governing tooth root development are distinct (Park et al., 2007; Zhang et al., 2015). Additionally, short root anomaly (SRA), caries, periodontitis, and trauma can cause tooth loss, affecting patients’ physical and mental health (Yu et al., 2021; Zhang et al., 2023). Dental implants have become widely uesd for tooth restoration; however, they lack true periodontal tissues between the implants and alveolar bone due to their biological inactivity. Moreover, the regeneration of a complete tooth remains challenging due to the complexity of tooth structures and the difficulties in guiding tooth eruption at the desired location. Therefore, it is imperative to develop technologies to regenerate bio-roots that closely resemble natural tooth roots.

Tooth root development involves complex process of epithelial-mesenchymal interactions, with various signaling pathways, such as Transforming Growth Factor β (TGFβ) and WNTs, being implicated in this process (Li et al., 2017). Bone Morphogenetic Proteins (BMPs), members of the TGFβ family, are well-known regulators of tissue development by modulating cellular activities, including proliferation, differentiation, and migration (Zhang and Que, 2020). Both epithelial cells and dental mesenchymal cells express BMP ligands and receptors (Thomadakis et al., 1999). BMP signals play important roles in the formation of the epithelial root sheath and the differentiation of odontoblasts and cementoblasts, suggesting that BMPs hold significant potential as bioactive factors for tooth root regeneration. This review focuses on the unique roles of BMP signaling in the development and regeneration of tooth roots.

## 2 BMP signaling pathway

The Bone Morphogenetic Proteins (BMPs) belong to the Transforming Growth Factor β (TGFβ) family of extracellular signaling proteins. The term “BMP class” has been used to refer to all named BMPs and Growth and Differentiation Factors (GDFs) in certain context (Gipson et al., 2020). Additionally, BMPs are characterized by their preferential signaling through a specific set of type I receptors, which ultimately activate transcription by phosphorylating of SMADs 1, 5 and 9.

Up to now, researchers have identified at least 20 BMP members that are structurally and functionally related across various species. Based on similarities in the amino acid sequence and receptor specificity, BMP ligands in most mammals can be classified into five different subcategories: BMP-2/4, BMP-5/6/7/8, BMP-9/10, BMP-12/13/14 (also known as GDF-5/6/7), and BMP-15 (Mueller and Nickel, 2012; Hinck et al. 2016; Yadin et al. 2016). BMPs can bind to their receptors only after they are cleaved by proprotein convertases, which convert them from their precursor form into the mature form.

The receptors involved in the BMP signaling pathway can be divided into two types, type I and typ (Heldin and Moustakas, 2016)e II (Gipson et al., 2020). Type I receptors include activin A receptor like type 1 (ACVRL1)/activin receptor-like kinase 1 (ALK1), activin A receptor type 1 (ACVR1)/ALK2, BMP receptor type 1A (BMPR1A)/ALK3, and BMPR1B/ALK6 (Hinck et al., 2016; Yadin et al., 2016; Nickel and Mueller, 2019). Type II receptors include BMP receptor type II (BMPR2), ACVR2A, and ACVR2B (Olsen et al., 2015). Different BMP subcategories have varying affinities for different type I and type II receptors. For example, BMP-2 and BMP-4 preferentially bind to BMPR1A and BMPR1B (Zhang et al., 2008; Morimoto et al., 2015). BMP-5/6/7/8 have a high affinity for ACVR1 and a low affinity for BMPR1A and BMPR1B (Ebisawa et al., 1999; Allendorph et al., 2006; Mueller and Nickel, 2012; Hinck et al., 2016; Yadin et al., 2016). BMP-9/10 primarily signal through ACVRL1, although BMP-9 also has affinity for ACVR1 and BMP-10 for BMPR1A (Mi et al., 2015; Tillet et al., 2018). BMP-12/13/14 preferentially interact with BMPR1B and have a low affinity for BMPR1A (Eliasson et al., 2008). BMP-15 binds to BMPR1A and BMPR1B, and can also form heterodimers with GDF-9, which signals through ALK-4/5/7 to activate SMAD2/3 (Chang et al., 2013; Yadin et al., 2016).

**FIGURE 1:**
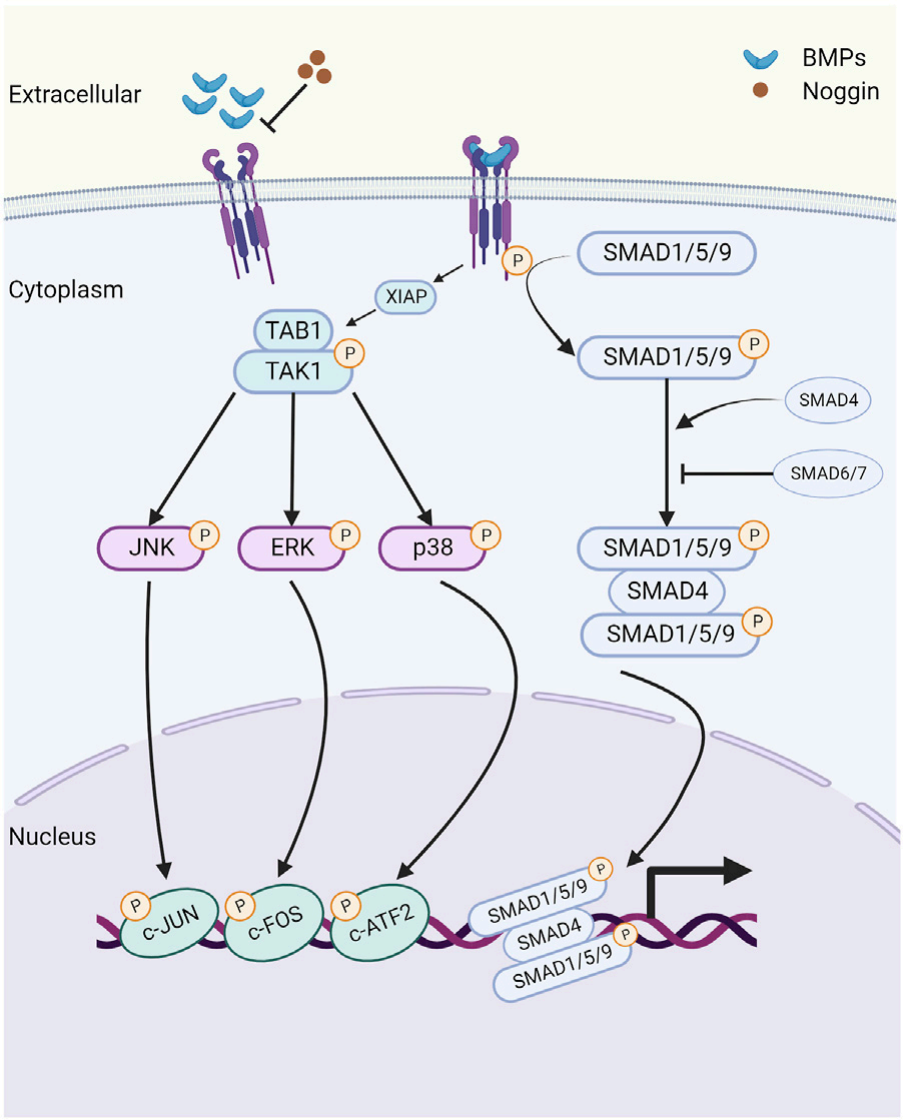
The schematic of the bone morphogenetic protein (BMP) signaling pathway. Created with BioRender.com. Initially, BMP ligands bind to type II receptors (BMPR2/ACVR2A/ACVR2B), which in turn recruit and phosphorylate type I receptors (BMPR1A/BMPR1B/ACVR1/ACVRL1). This phosphorylation event leads to the activation of SMAD1/5/9 proteins, which then form a complex with the common SMAD (SMAD4) and transduce signals to the nucleus, modulating the expression of downstream target genes, including Id1 and Id2. Extracellular antagonist Noggin suppresses BMP signaling by inhibiting the binding of BMP ligands with their corresponding receptors. Additionally, inhibitory SMAD6 and SMAD7 prevent the phosphorylation and nuclear translocation of SMAD1/5/9 proteins. Furthermore, noncanonical BMP signaling transduces through phosphorylated TAK1 (pTAK1). BMPRI interacts with the TAK1-TAB1 complex via X-linked inhibitor of apoptosis (XIAP), leading to the phosphorylation and activation of downstream mitogen-activated protein kinases (MAPKs), including p38, ERK1/2, and JNK. Consequently, these activated kinases translocate to the nucleus, where they phosphorylate and activate ATF2, c-JUN, and c-FOS, thus regulating the transcription of downstream target genes.

The canonical BMP signaling pathway involves the participation of type I and type II receptors and SMAD complexes (Yu et al., 2005). BMP ligands initially bind to type II receptors, which recruit and phosphorylate type I receptors (Shi and Massague, 2003). Subsequently, the ligand-receptor complex phosphorylates the intracellular molecules SMADs 1, 5, and 9 (SMAD1/5/9) at two serine residues in the C terminus. Following phosphorylation, SMAD1/5/9 (p-SMAD1/5/9) forms a complex with SMAD4, known as the common SMAD. This complex translocates to the nucleus, where it directly regulates the transcription of downstream target genes, including Id2 (Shi and Massague, 2003) (Figure 1).

In addition to SMAD-mediated signaling activation, BMPs can also signal through a non-canonical pathway, which includes the activation of type I and type II receptors. This pathway operates independently of the SMAD proteins and instead relies on components of the MAPK and PI3K-AKT pathways (Brown et al., 2005). Previous research has established that BMPRIA can interact with the TAK1-TAB1 complex through the E3 ubiquitin-protein ligase X-linked inhibitor of apoptosis (XIAP), leading to TAK1 activation (Yamaguchi et al., 1999). Subsequently, TAK1 phosphorylates and activates downstream MAPKs, including p38, ERK1/2, and JNK (Shibuya et al., 1996). As a result, these activated molecules translocate to the nucleus, where they phosphorylate and activate substrates such as ATF-2, c-Jun, and c-Fos, thereby regulating the transcriptions of downstream target genes.

There are three categories of BMP signaling inhibitors. (1) Extracellular antagonists, including noggin, chordin, follistatin, Tsg, and members of the Dan family (Dan, Cer, Coco, Gremlin, USAG-1, sclerostin) (Yanagita, 2005). (2) Intracellular inhibitors, such as SMAD6 and SMAD7, which negatively regulate BMP signaling through various mechanisms. These mechanisms include the formation of inactive complexes with SMADs, promotion of the ubiquitination and degradation of BMP receptors, or direct binding to DNA to repress transcription (Hata et al., 1998; Hanyu et al., 2001). (3) Regulation within the nucleus. The levels of SMAD transcriptional activity are controlled by numerous coactivators and corepressors (Hill, 2016).

## 3 Processes of tooth root development

**FIGURE 2:**
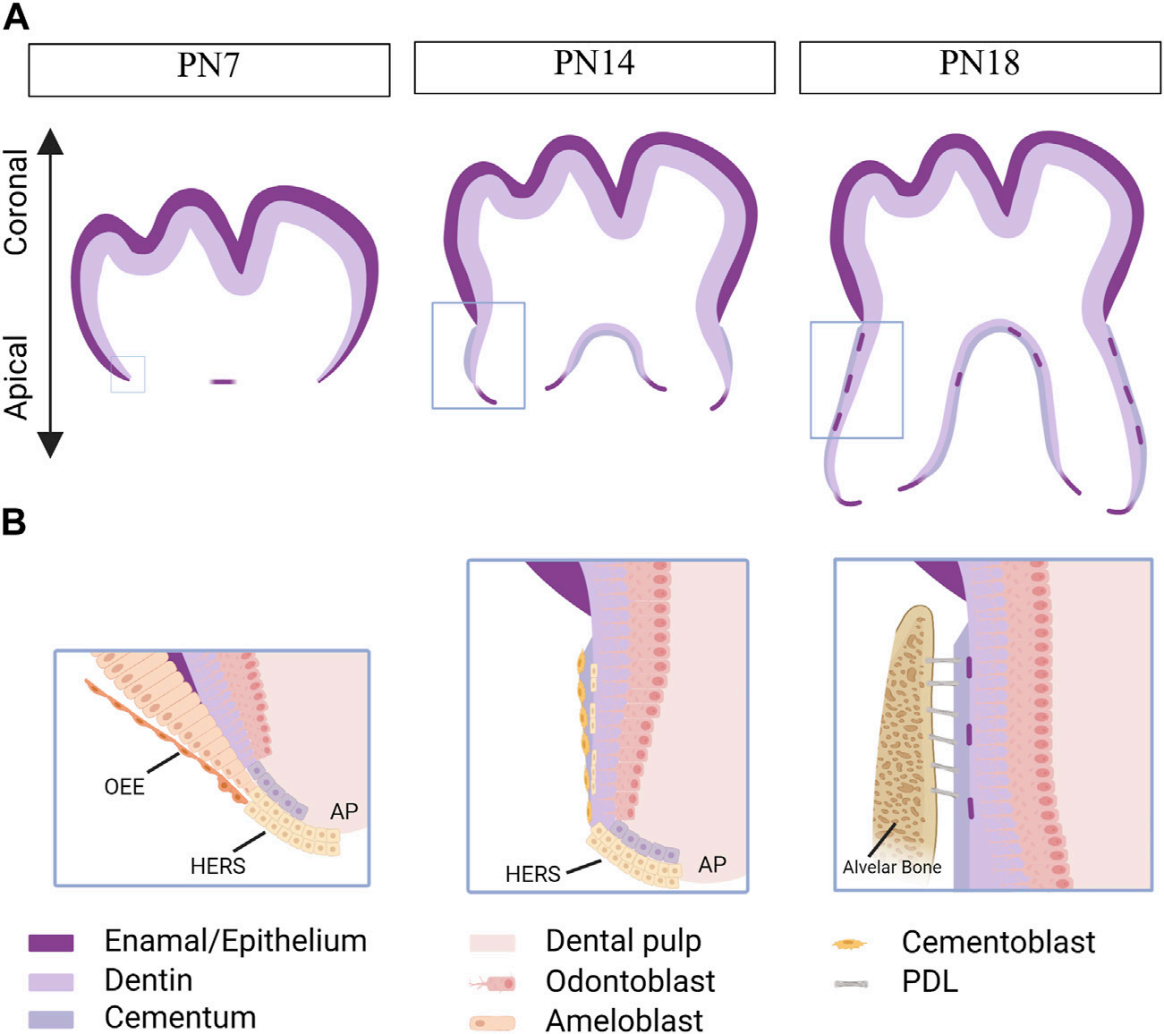
Tooth root development in mice. Created with BioRender.com. **(A)** Schematics of root development in mice at postnatal day (PN) 7, 14 and 18. As the tooth root develops, its length increases, and root dentin, cementum, and the periodontal ligaments (PDLs) are observed within the alveolar bone. Boxed areas in Fig. A represent regions shown in detail in Fig. **(B)**. At PN7, the outer enamel epithelium and inner enamel epithelium unite at the cervical loop, giving rise to a bilayer structure known as Hertwig’s epithelial root sheath (HERS). At PN14, HERS induces the differentiation of apical papilla cells into odontoblasts, which then secret extracellular matrix to form root dentin. Following the disintegration of HERS, dental follicle cells come into contact with the newly formed root dentin, initiating their differentiation into cementoblasts responsible for cementum formation. By PN18, root development is fully completed, and the tooth has erupted, although the closure of the apical foraman remains incomplete. The HERS migrates toward the periodontal ligament and becomes the epithelial rests of Malassez. OEE, outer enamel epithelium. AP, apical papilla.

Similar to tooth development, tooth root development is a complex process involving epithelial-mesenchymal interactions (Balic and Thesleff, 2015). After crown formation, the outer enamel epithelium joins with the inner enamel epithelium at the cervical loop, forming a bilayer structure called Hertwig’s epithelial root sheath (HERS) (Ten Cate, 1996). HERS plays a dual role as both a barrier and a signaling center, extending apically to enclose the dental papilla and separate it from the dental follicle. The apical papilla, dental follicle, and HERS are considered an inseparable integrity known as the developing apical complex (DAC). HERS induces the differentiation of dental papilla cells into odontoblasts, which then form the primary hard tissue of root dentin. However, HERS is a transient structure that becomes disintegrated after the formation of root dentin. The disintegration of HERS facilitates the contact of dental follicle cells with the root dentin, initiating the differentiation of dental follicle cells into cementoblasts to form cementum (Figure 2). Therefore, the formation, extension, and degeneration of HERS are critical factors in determining the length, shape, and number of tooth roots (Bosshardt et al., 2015). Meanwhile, the signals from the apical papilla regulates the fate of the dental follicle by sustaining the stemness of dental follicle during tooth root development (Wu et al., 2018).

Moreover, HERS functions as a signaling center by producing growth factors, such as Sonic Hedgehog (SHH), that contribute to maintaining the function of epithelial cells and inducing the differentiation of apical papilla. Multiple outcomes for HERS have been proposed, including undergoing apoptosis, transforming into the epithelial rests of Malassez (ERM), and undergoing epithelial-mesenchymal transformation to differentiate into cementoblasts (Huang et al., 2009).

Although the roles of epithelial-mesenchymal interactions during root development are not fully understood, multiple signaling molecules, such as TGF-β/BMP, WNTs, SHH and fibroblast growth factors (FGFs), have been implicated in this process (Li et al., 2017)

## 4 Roles of BMP signaling in tooth root development

BMP signaling is involved in different stages of tooth root development. In this context, we will delve into the root development of the first mandibular molar in mice as an illustrative example. By postnatal day (PN) 3, crown development is completed, marking the onset of tooth root development. Subsequently, the location of active BMP/SMAD signaling gradually shifts from the crown to the root.

Between PN3 and PN5, concurrent with tooth root development, stem cells crucial for tooth root development progressively emerge, accompanies by evident BMP/SMAD signaling within the dental pulp. Progressing to PN6-PN8, the HERS gradually forms and extends towards the root, simultaneously leading to the formation of the DAC. The distribution of BMP/SMAD signaling gradually extends from the coronal to the apical region, forming associations with blood vessels within the dental pulp and the DAC zone.

During the PN9-PN14 phase, there is swift elongation of the tooth root alongside rapid mineralization of tooth root dentin. At this stage, robust BMP/SMAD signaling is notably detected within the root pulp and the apical region. As PN15-PN18 approaches, the rate of tooth root elongation slows down, indicating near-completion of root length development, even though the apical foramen remains wide. During this peiod, the activity of BMP signaling gradually weakens, but intensified in the apical region and the root pulp.

Progressing to PN19-PN21, the molar gradually erupts into the oral cavity, achieving occlusion. Simultaneously, the narrow apical foramen gradually forms. Notably, BMP/SMAD signaling is only activated along blood vessels in the root pulp (Wang et al., 2021). For a clearer representation of the expression of BMP ligands and receptors in tooth root development, we have summarized this information in Table 1.

**TABLE 1:** The expressions of BMP ligands and receptors in developing apical complex.

	HERS	Apical papilla	Dental sac
BMP-2	+	+	+
BMP-4	+	+	+
BMP-5	+	+	+
BMP-6	unknown	unknown	+
BMP-7	+	+	+
BMP-8	unknown	unknown	unknown
BMP-9	+	+	+
BMP10	unknown	unknown	unknown
GDF-5/6/7	−	unknown	+
ACVRL1	unknown	unknown	unknown
ACVR1, BMPR1B, BMPR2	+	+	+
BMPR1A	+	+	+

+, positive; −, negative.

Additionally, studies have reported that targeted downregulation of BMP signaling in the epithelium using keratin 14-Noggin transgenic mice leads to reduced sizes of the first and second maxillary molars, altered crown patterns, and failed formation of multiple roots (Plikus et al., 2005).

Collevetively, BMP signaling plays important roles throughout tooth root development (Table 2).

**TABLE 2:** Functions of BMP signaling in the development of tooth roots.

Gene	Model	Defect	Reference
*Bmp2*	*Osx-*Cre	Short root	(Rakian et al., 2013)
*Bmp2*	*Osx*-Cre^ERt^	Short root	(Guo et al., 2015)
*Bmp2*	3.6 kb Col1a1-Cre	Short root	(Yang et al., 2012)
*Bmp2*; *Bmp4*	*Dmp1*-Cre	Short root	(Jani et al., 2018)
*Bmp2*; *Bmp4*	*K14-*Cre	Short root	(Mu et al., 2021)
*Bmp9*	Null	Short root	(Huang et al., 2019)
*Acvr1*	*Osx-*Cre	Short root	(Zhang et al., 2019)
*Bmpr1a*	*Krt5-rtTA; tetO-*Cre	ectopic cementum-like structures	(Yang et al., 2013)
*Bmpr1a*	*Krt14-rtTA; tetO-*Cre	Short root	(Li et al., 2015)
*Bmpr1a*	*Osx-*Cre	Short root	(Omi et al., 2020)
*Smad4*	*Osr2-Ires-*Cre	Short root	(Li et al., 2011)
*Smad4*	*Krt14-*Cre	NO root	(Huang et al., 2010)
*Smad4*	*Krt14-rtTA; tetO-*Cre	longer crown/Short root	(Li et al., 2015)
*Smad4*	*OC-*Cre	Short root	(Gao et al., 2009)
*Noggin*	KRT14-promoter	Short root/single root	(Plikus et al., 2005)

### 4.1 Roles of BMP ligands in tooth root development

#### 4.1.1 BMP-2

**FIGURE 3:**
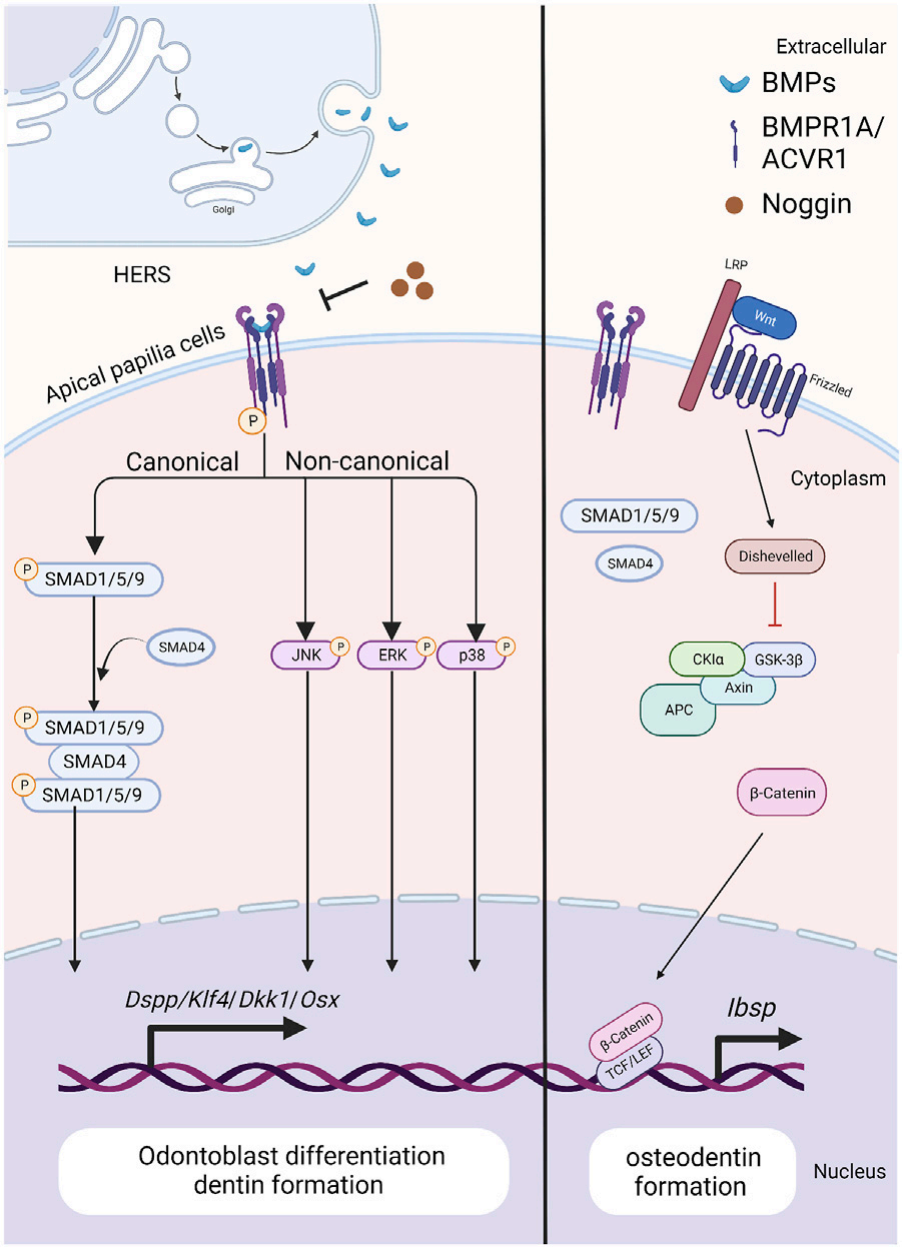
The BMP signaling pathway in apical papilla cells during tooth root development. Created with BioRender.com. BMP ligands (such as BMP2/4) bind to BMP receptor type II and BMP receptor type I (BMPR1A/ACVR1) complexes, thus activating canonical and noncanonical BMP signaling and modulating the expression of downstream target genes, including *Dspp*, *Klf4, Dkk1* and *Osx*. As BMP signaling is blocked by ablation of BMP ligands or BMP receptors, the expressions of WNT antagonists, Dkk1 and Sost, are downregulated, but β-catenin and *Ibsp* is upregulated. As a result, it specifies the fate of dental pulp and promotes the differentiation of apical papilla cells into odontoblasts. Noggin acts as an extracellular antagonist, suppressing BMP signaling leads to reduced size in the first and second maxillary molars, altered crown patterns, and failed formation of multiple roots.

During tooth root elongation, *Bmp2* is detected obviously in odontoblasts, dental pulp cells, and osteoblasts, and weakly in ameloblasts, as demonstrated by *in situ* hybridization (Yamashiro et al., 2003; Kemoun et al., 2007). While the BMP-2 derived from ectoderm is not as potent as that originating from dental mesenchyme, it nevertheless plays a role in sustaining epithelial-mesenchymal interactions during tooth root development (Mu et al., 2021) (Figure 3). Multiple studies have demonstrated the critical role of BMP-2 in dentin development, where it determines the fate of dental mesenchymal stem cells (DMSCs), drives their differentiation into odontoblasts, and simultaneously enhances the expression of genes associated with tooth development (Yang et al., 2012; Guo et al., 2015; Jani et al., 2018). Deletion of *Bmp2* in *Osterix*-expressing mesenchymal progenitor cells lead to shortened roots and abnormal odontoblast differentiation (Rakian et al., 2013). The root dentin of *Osx-*Cre*; Bmp2* ^*fl/fl*^ mice exhibits notable dysplasia, with odontoblasts entrapped within the matrix, resulting in the formation of osteodentin. In these mice, a significant amount of osteodentin forms at the apical region of the root pulp, suggesting cell autonomous alterations in dental mesenchyme. These results underscore the requirement of both ectoderm-derived and mesenchyme-derived BMP-2 for odontoblast differentiation and root formation.

#### 4.1.2 BMP-4

**FIGURE 4:**
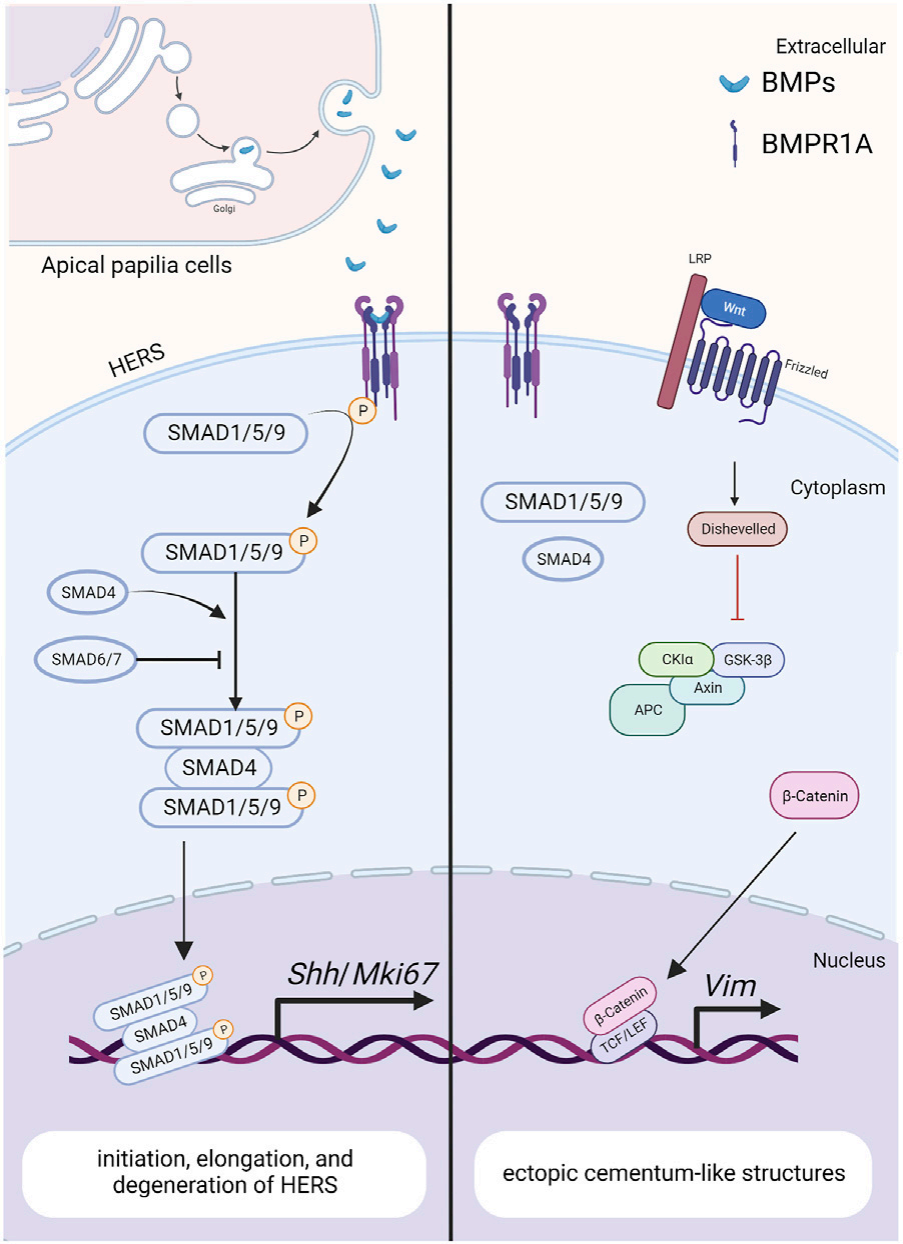
The BMP signaling pathway in HERS during tooth root development. Created with BioRender.com. BMP ligands (such as BMP2/4) bind to BMP receptor type II and BMPR1A complexes, thereby activating canonical BMP signaling and modulating the expression of downstream target genes, including *Shh* and *Mki67*. These genes play crucial roles in regulating the initiation, elongation and degeneration of HERS. As a downstream target of BMP signaling pathway in HERS, SHH also promotes the differentiation of root odontoblasts through the epithelial-mesenchymal interactions. The antagonistic interaction between BMP and Wnt signaling pathways further promotes the epithelial-mesenchymal transition during tooth development.

*Bmp-4* is detected within the epithelial cells of HERS, preodontoblasts surrounding HERS, and odontoblasts during tooth root development, as revealed by *in situ* hybridization (Yamashiro et al., 2003). Similar to BMP-2, the ectoderm-derived BMP-4 is required for the degeneration of HERS and differentiation of root odontoblasts, where they play redundant roles (Mu et al., 2021). Hosoya et al. demonstrated that the length of HERS and the number of PCNA-positive cells are significantly enhanced when treated with Noggin beads, compared with those treated with Bmp4 beads (Hosoya et al., 2008) (Figure 4). Furthermore, *BMP4* mutations have been reported in patients with conditions such as tooth agenesis, root malformation, and taurodontism (Kantaputra et al., 2022). Collectively, these findings indicate that BMP-4 prevents the elongation of HERS by inhibiting cell proliferation.

Moreover, BMP-4 can act as a downstream mediator, directly promoting odontoblast differentiation and tooth root formation. Ablation of Ring1a/b in dental mesenchyme results in shortened teeth and impaired odontoblast differentiation, accompanied by elevated *Bmp4* expression (Lapthanasupkul et al., 2012). Similarly, conditional disruption of *Fam20c* in both dental epithelium and dental mesenchyme leads to pronounced reduction in BMP-4 and phosphor-Smad1/5/9 levels, resulting in severe shortened roots and abnormal odontoblast differentiation. This disruption also correlates with decreased expression of downstream targets, including *Osterix* and Krüppel-like factor 4 (Klf4) (Li et al., 2022). These findings provide additional evidence supporting the significance of BMP-4 in influencing odontoblast differentiation and root formation at precise concentrations.

#### 4.1.3 Other BMPs

At PN15 in the first molar of mice, BMP-6 protein is detectable in cells around the apical region of the dental root and within the periodontium, as confirmed by immunohistochemistry (Oralova et al., 2014). A gradual increase in *Bmp6* expression is observed from postnatal days 1–11 in rats, as demonstrated by RT-qPCR. Moreover, BMP-6 promotes alveolar bone formation at the crypt’s base and contributes to tooth eruption (Wise et al., 2011). However, the specific role of BMP-6 in tooth root development remains ambiguous, mainly due to the limited influence of tooth root on the eruption process. Furthermore, mutations of *BMP6* in humans and mice lead to massive iron overload (Meynard et al., 2009). Studies have revealed the inhibitory effects of iron intoxication on the processes of dentine mineralization (De Lucca et al., 1999). However, the roles of BMP-6 in tooth root formation have not been elucidated in detail, although BMP-6 may promote dentine and alveolar bone mineralization by regulating iron metabolism.

*Bmp7* is detected in dental pulp, preodontoblasts, ameloblasts, and cells within the periodontium during tooth root development, as indicated by *in situ* hybridization (Yamashiro et al., 2003; Kemoun et al., 2007). Nevertheless, the specific roles of BMP-7 in tooth root formation have not been extensively explored.

*Bmp9*-KO mice display characteristics such as short roots and thinner dentin, resembling the phenotypes observed in other mice with blocked BMP signaling pathways (Huang et al., 2019). Nevertheless, the underlying molecular mechanisms through which BMP-9 influences tooth root development warrant further investigation.

Reverse-transcriptase polymerase chain-reaction (RT-PCR) results show that *Gdf5/6/7* are expressed within bovine dental follicles surrounding the developing roots (Morotome et al., 1998). *In situ* hybridization results show that *Gdf5/6/7* mRNA are expressed in osteoblasts, cellular cementum, and large numbers of cells in the developing periodontal ligament of three-week-old rats. The expressions of GDFs in cells of periodontal tissue are downregulated after the completion of root formation. However, at 36 weeks, the expressions of GDFs are not visible in periodontal tissue. During the observation time, signals for GDFs are not detected in HERS (Sena et al., 2003). These results suggest that GDF-5/6/7 may be involved in the formation of periodontal tissues, but the roles of GDF-5/6/7 in root development need to be further investigated.

For other BMP ligands, i.e., BMP-5/8/10/15, the expressions and roles in tooth root development have not been reported so far.

Although various BMP ligands are involved in tooth root formation, the mechanisms of BMPs regulating tooth root development are not fully elucidated.

### 4.2 Roles of BMP receptors in tooth root development

#### 4.2.1 ACVR1

*In situ* hybridization and Northern Blot results show that *Acvr1* is expressed in dental pulp cells in adult rat, bovine, and human (Cheifetz, 1999). While, the ACVR1 protein is observed within HERS, dental pulp, dental follicle, and cells in PDL through immunohistochemistry staining (Kemoun et al., 2007). Disruption of *Acvr1* in dental mesenchymal cells using *Osx*-Cre leads to shortened roots, thinner dentin, and osteodentin formation, associated with a shift in cell fate from odontoblasts to osteoblasts. Notably, the expressions of WNT antagonists, Dkk1 and Sost, are downregulated, whereas β-catenin is upregulated in incisors of *Acvr1* ^*fx/−*^ *; Osx-*Cre; *R26R/+* mice, suggesting that BMP signaling could interact with Wnt signaling to control root formation (Zhang et al., 2019) (Figure 3). Moreover, the phenotypes of *Acvr1* cKO are similar to those observed in *Bmp2*-cKO*; Sp7*-Cre-EGFP mice (Rakian et al., 2013), indicating that ACVR1 may serve as the preferential receptor for BMP-2 during tooth root development.

#### 4.2.2 BMPR1A

When apical papilla cells commit to differentiating into odontoblasts, BMP/SMAD signaling becomes activated. Disruption of *Bmpr1a* in the root mesenchyme using *Gli1-CreER* prior to root formation results in impaired root development (Feng et al., 2017) (Figure 3). Specifically, these mice exhibit the absence of molars’ roots, despite that the crown-to-root transition has been completed. Therefore, BMPR1A-mediated BMP signaling activity is required for driving the odontogenic differentiation of apical papilla cells and initiating the process of tooth root formation.

Loss of *Bmpr1a* in dental mesenchyme using *Osx*-Cre after birth leads to shortened molar roots and reduced dentin thickness by PN21 (Omi et al., 2020). To further dissect the functions of BMPR1A-mediated BMP signaling in tooth root development, *Bmpr1a* cKO mice are generated with constitutive activation of Smad1/5/9 signaling. The resulting compound mutants successfully rescue thinner crown dentin in *Bmpr1a* cKO mice, yet the conditions of short roots and abnormal root dentin formation remain unchanged. These findings suggest that BMPR1A-mediated canonical BMP signaling primarily influences crown dentin formation, while non-canonical BMP signaling likely plays a critical role in root dentinogenesis and development (Figure 3).

Ectopic cellular cementum-like structures are observed on tooth crowns in *Krt5-rtTA; tetO*-Cre mice, with Cre activity induced at E14.5. This observation suggests that BMP signaling in crown epithelia prompts the transition of epithelial differentiate into the root lineage and facilitates epithelial-mesenchymal transition. Concomitant depletion of β-catenin (*Krt5-rtTA*; *tetO-*Cre*; Alk3* ^*fl*/*fl*^ *; Ctnnb1* ^*fl*/*fl*^) rescues the ectopic cementogenesis caused by *Bmpr1a* depletion. This finding underscores the antagonistic interaction between BMP and Wnt signaling in epithelial cells, which governs the transition from crown to root formation at the onset of tooth root development (Yang et al., 2013). (Figure 4)In addition, disruption of *Bmpr1a* in dental epithelium using *Krt14-rtTA; tetO-*Cre results in shortened roots without the formation of ectopic cellular cementum-like structures (Li et al., 2015). This suggests that BMP signaling assumes distinct roles in different epithelial cell types, and the BMPR1A-mediated BMP signaling cascade regulates the transitional process of epithelial cells during tooth root development.

#### 4.2.3 Other BMP receptors

At PN6 and PN13, during tooth root formation, BMPR1B and BMPR2 are detected in HERS, DF, and dental papilla cells by immunohistochemistry (Kemoun et al., 2007). These results indicate that BMPR1B and BMPR2-mediated BMP signaling cascade may be involved in the formation of tooth root and periodontal tissues, but the roles of BMPR1B and BMPR2 in root development need further investigation. Given the cross-interaction between BMP ligands and BMP receptors, knocking out or knocking down specific BMP receptors provides a promising approach to investigate the roles of BMP signaling in tooth root development. Currently, research mostly focuses on ACVR1 and BMPR1A, while there are few reports on other BMP receptors, i.e., ACVRL1, ACVR2A, and ACVR2B. The expressions and roles of these receptors in tooth root development have not been reported so far.

### 4.3 Roles of Smad4 in tooth root development

As a central intracellular mediator of the canonical BMP signaling pathway, Smad4 plays a crucial role in regulating tooth root development. Conditional inactivation of *Smad4* in dental epithelium using *Krt14-rtTA; tetO*-Cre during tooth root development leads to abnormal dentin formation and shortened roots (Li et al., 2015). In these mice, the enlargement of HERS without elongation disrupts tooth root development at the initiation stage. Furthermore, the expressions of Shh within dental epithelium and Nfic within dental mesenchyme are significantly decreased, indicating that Smad4-dependent BMP signaling regulates the elongation of HERS and the differentiation of DMSCs via SHH signaling, thereby initiating tooth root development (Huang et al., 2010). Given the role of BMPR1A-mediated BMP signaling cascade in epithelial stem cell transition, it is plausible that the BMP-BMPR1A-SMAD4-SHH-Gli1 signaling cascade in epithelia cells orchestrates the formation of HERS and the differentiation of apical papilla during root initiation (Li et al., 2015). (Figure 4)

Furthermore, Smad4-dependent BMP signaling in dental mesenchyme plays a critical role in tooth root development. Ablation of *Smad4* in odontoblasts using *Osteocalcin*-Cre leads to shortened roots, impaired odontoblast differentiation, and the formation of osteodentin (Gao et al., 2009). This defect is similarly observed on the lingual side of incisors (root analogue) in mutant mice. Moreover, the phenotypes of *Smad4* cKO closely resemble those observed in *Acvr1* cKO and *Bmp2*-cKO*; Sp7*-Cre-EGFP mice (Rakian et al., 2013), indicating that BMP2-ACVR1-SMAD4 signaling cascade may orchestrate odontoblast differentiating to form root dentin. Notably, it is worth mentioning that SMAD4 also serves as a central intracellular mediator of TGF-β signaling pathway. Furthermore, this phenotype parallels what is observed in *Tgfbr2* mutant mice, highlighting the need for extensive research into the role of Smad4-dependent BMP signaling in root dentinogenesis. Interestingly, the *OC-*Cre; *Smad4* ^*fl/fl*^ mice exhibit enlarged ERM within the periodontal ligament, and they even form odontogenic keratocysts. This finding indicates that the disruption of Smad4-dependent BMP in odontoblasts results in the defective outcomes of HERS, emphasizing the importance of mesenchymal-epithelial interactions.

## 5 Can BMP signaling be a target for tooth root regeneration therapy?

Tooth loss is a prevalent dental condition often caused by caries, periodontitis, trauma, and SRA. Nowadays, biologically inactive dental implants have become widely utilized for tooth restoration (Chen et al., 2021). However, they lack a periodontal connection with the alveolar bone, resulting in an inability to evenly distribute chewing pressures. Additionally, due to the absence of pulp tissue, the implants fail to sense changes in temperature or pressure. Compared with natural tooth roots, dental implants exhibit significantly higher compressive strength, modulus of elasticity, and torsional force (Gao et al., 2016). Therefore, it is imperative to develop technologies for regenerating bio-roots. Notably, BMP signaling is recognized to regulate tooth number. USAG-1 (Uterine sensitization-associated gene-1) functions as a BMP antagonist, and its deficiency leads to the formation of supernumerary teeth by enhancing BMP signaling. Therefore, targeting BMP signaling by anti-USAG-1 therapy holds promise for whole tooth regeneration (Murashima-Suginami et al., 2021). Nevertheless, the complexities of tooth structures and challenges in guiding tooth eruption at the desired location have made whole tooth regeneration a formidable task. As an alternative, focusing on regenerating tooth roots alone could emerge as a viable strategy in the future.

Utilizing tissue engineering for root regeneration presents an avenue to uphold the natural functionality of teeth in a more physiological manner. This approach involves the regenerating of a bioengineered tooth root (bio-root) along with its associated periodontal tissues, providing a biological alternative to conventional dental implants.

### 5.1 Root-shaped TDM

To simulate the natural morphology of a tooth root, researchers have developed a treated dentin matrix (TDM), which serves as a natural 3D graft material derived from dentin matrix (Guo et al., 2009). It is reported that TDM maintains the physical and chemical properties of dentin, with dentin tubules still intact and its chemical composition remaining unchanged (Li et al., 2011). Comprising 70% minerals, 20% organic components, and 10% water (by weight), TDM’s composition closely resembles that of dentin. Importantly, the organic components of TDM, including DSPP, DMP1, and other growth factors, retain their bioactivity even after cryopreservation (Jiao et al., 2014).

In an intriguing study, Jin and colleagues transplanted rat TDM together with rat dental follicle stem cells (DFSCs) into the alveolar fossa microenvironment, resulting in the formation of a tooth root (Guo et al., 2012). Subsequent studies have highlighted that the indispensability of this specific environment for tooth root formation, as DFSCs fail to generate a tooth root when transplanted into the skull or omental pockets.

Although autologous TDM is limited in origin compared with xenogenic TDM, it demonstrates better capabilities in promoting dentin regeneration and odontoblast differentiation. In contrast, xenogenic TDM poses challenges due to transplant rejection (Li et al., 2021). Overall, TDM proves to be a suitable graft material for future bio-root regeneration. However, effective immunomodulation methods require further exploration.

TDM can be manufactured in various shapes, including sheets, slices, root-shaped forms, barrier membranes, and press-fitted configurations, tailored to the specific defect shape (Chen et al., 2015; Grawish et al., 2022). Notably, the regenerated mineralized matrix closely mirrors the original TDM shape, underscoring the potential to achieve tooth roots with diverse configurations by utilizing prefabricated TDM shapes (Guo et al., 2012). Studies have reported that computer-aided design (CAD) and finite element analysis (FEA) can be employed to optimize scaffold shape and size, enhancing biomechanical strength and achieving desirable shapes of the regenerated mineralized matrix (Luo et al., 2015). Although shape-optimized TDM scaffolds hold promise for bio-root regeneration, further studies are warranted to investigate their clinical applications and outcomes in humans.

To our knowledge, no standard protocol for demineralization has been established for tooth root regeneration (Huang et al., 2019). Therefore, optimizing the concentration and pH of the demineralizing agent, as well as adjusting the exposure time, are essential in developing the protocol. Previous studies have recommended immersing TDM in PBS containing penicillin and streptomycin for 72h, followed by rinsing with sterile deionized water for disinfection (Bakhtiar et al., 2017; Chen et al., 2017). Nevertheless, there remains a potential for immune response and patient infections. Autoclaving, a widely used sterilization method in hospitals and labs, provides a simple, rapid, and efficient option (Chang et al., 2020). Nonetheless, for clinical applications, further research is needed to determine the appropriate disinfection methods and standards for TDM.

### 5.2 Seed cells types

Stem cells play a crucial role in tissue engineering, particularly in achieving the regeneration of an ideal root (Chen et al., 2022). Current researches focus on understanding the behaviors of different types of stem cells. DMSCs are somatic stem cells that can be isolated from various dental tissues, including DFSCs, dental pulp stem cells (DPSCs), stem cells from human exfoliated deciduous teeth (SHED), periodontal ligament stem cells (PDLSCs), stem cells from the apical papilla (SCAPs), gingival mesenchymal stem cells (GMSCs), alveolar bone-derived mesenchymal stem cells (ABMSCs), and so on. Among them, DFSCs, DPSCs, SHED, PDLSCs (Tomokiyo et al., 2019) and SCAPs have been reported for using in root regeneration, while the potential applications of others remain promising, although not yet extensively studied.

DFSCs are a type of dental mesenchymal stem cells that can be isolated from extracted third molars with minimal damage to subjects and without ethical controversy. Compared to DPSCs and PDLSCs, DFSCs demonstrate a superior capacity for proliferation and osteogenic-related differentiation (Gan et al., 2020). While the regeneration of periodontal tissue remains a challenge in bio-root research and applications, DFSCs derived from the dental follicle, which contributes to the development of periodontal tissues including alveolar bone, cementum, and PDL, hold promise. In addition, DFSCs can differentiate into odontoblasts to regenerate dentin, suggesting their potential to contribute to the formation of multiple important periodontal tissues along with dentin in the future. Zhou et al. have reviewed the clinical application potentials of DFSCs in tooth regeneration (Zhou et al., 2019). Consequently, DFSCs have become a focal point of research for bio-root regeneration. Researchers have conducted studies where they transplanted DFSCs together with TDM into the alveolar bone of mini-swine and Sprague-Dawley rats, as well as performed subcutaneous transplantation in rat and nude mice, leading to the formation of a tooth root (Guo et al., 2012).

Similar to DFSCs, DPSCs are also multipotential stem cells and can serve as seed cells for bio-root regeneration. In order to achieve a more comprehensive bio-root regeneration encompassing both the periodontium and pulp-dentin complex, a sandwich structure consisting of hDPSC/TDM/Matrigel is developed and transplanted subcutaneously in nude mice for a duration of 3 months (Meng et al., 2020). Due to multipotent differentiation properties of DPSCs, a pulpo-dentinal complex forms within the pulp cavity of TDM, while a periodontium-like dense connective tissue is observed outside the TDM. Therefore, DPSCs can be utilized as seed cells for the regeneration of the bio-root, and the sandwich structure composed of cells/TDM/cells shows potential for tooth root regeneration.

SHED is another important category of DMSCs, exhibiting a notably higher proliferative potential compared to other types. In a series of experiments, researchers separately transplanted SHED along with TDM into the subcutaneous tissue and jawbone of nude mice and rats to detect the regenerative capacity of SHED in bio-root (Yang et al., 2019). Following an 8-week implantation period, distinct regeneration of fresh dentin and periodontal tissues was observed. These findings highlight the promising potential of SHED as seed cells for tooth root regeneration, suggesting plausible clinical applications.

Compared with DMSCs, adipose-derived stromal/stem cells (ASCs) have a wide range of sources and have been used in tissue engineering for many years. Recent study reports that regenerated dentin-like, pulp-like, and periodontal fiber-like tissues are found after transplanting ASCs with TDM into nude mice for 8 weeks (Yuan et al., 2022). This finding indicates that ASCs can be used as potential seed cells for bio-root regeneration, while further optimized researches are needed.

Stem cells play a crucial role in maintaining tissue homeostasis and facilitating tissue repair. The mobilization and homing of these cells provide an alternative approach for tissue regeneration. It is reported that successful root regeneration, encompassing PDL like tissues and cementum, has been achieved using autologous PRF and allogeneic TDM. This success is attributed to the facilitation of homing and differentiation of both PDLSCs and Bone marrow stem cells (BMSCs). Nonetheless, the prospect of tooth root regeneration through endogenous mobilization, homing, and differentiation of stem cells remains a promising approach that needs to be further investigated.

### 5.3 BMPs can be used as bioactive factors for tooth root regeneration

Biomolecules constitute a crucial component of tissue engineering-mediated root regeneration. Although research on utilizing BMPs to regenerate bio-roots is still relatively uncommon, these proteins hold significant potential as bioactive factors for tooth root regeneration. This potential is based on the role of BMP signaling in various stages of tooth root development and its ability to promote the differentiation of DMSCs into odontoblasts, cementoblasts, PDL cells, and osteocytes, contributing to the formation of dentin and periodontal tissues.

It has been reported that a biomimetic approach involving the incorporation of two crucial odontogenic growth factors (TGF-β1 and BMP-4) into the aforementioned TDM, which embodies a spatial interface gradient, facilitates the formation of a functional enthesis to promote bio-root regeneration (Chen et al., 2020). In a rat subcutaneous transplantation study, researchers transplanted a sandwich-like composition composed of TDM interlaid between the innermost growth factors and the outermost DFSCs. The synergistic activation of DFSCs’ proliferation, migration, and osteoinductivity by TGF-β1/BMP-4 has been demonstrated through increased expression of cell adhesion related proteins and osteogenic markers. This incorporation of TGF-β1/BMP-4 with TDM and DFSCs presents a feasible strategy for bio-root regeneration. Nevertheless, further studies are required to explore its clinical applications in humans.

Biomaterial scaffold-based bioengineered tooth roots have shown promise as a prospective approach for treating tooth loss. However, despite efforts to replicate the mechanical integrity of a native tooth root, the bio-root still faces limitations, including the challenge in achieving long-term functional stability due to the lack of an effective cervical seal. Targeting the BMP signaling pathway could offer potential solutions for enhancing tooth root regeneration therapy.

## 6 Conclusion

It is evident that BMP signaling regulates the tooth root development by promoting the formation of HERS and the differentiation of odontoblasts and cementoblasts. Both ectoderm-derived and mesenchyme-derived BMP signaling are required for odontoblast differentiation and root formation. However, the mechanisms by which BMPs exert their functions are still largely unknown. For example, the reason behind the formation of massive osteodentin at the apical region of the root pulp when blocking BMP signaling in mice is still unclear. Additionally, the receptors that mediate the signaling pathway in the regulation of cell fate autonomous alterations of dental mesenchyme remain unknown. Furthermore, while the crucial roles of BMP signaling in periodontal tissues can be leveraged for the regeneration of bio-roots to treat tooth loss, research on utilizing BMPs to regenerate bio-roots is still relatively uncommon. While the task of complete tooth regeneration remains challenging, stimulation of third dentition by activating BMP signaling, as akin to anti-USAG-1 therapy, holds promise as a viable strategy in the future. Overall, targeting the BMP signaling pathway could offer potential solutions for enhancing tooth root regeneration therapy.
